# Double gallbladder malformation with duodenal ulcer stricture leading to common bile duct dilation: a case report

**DOI:** 10.3389/fmed.2026.1856010

**Published:** 2026-07-02

**Authors:** Rui-Biao Fu, Xiao-Feng Dong

**Affiliations:** Department of Hepatobiliary, Pancreatic and Spleen Surgery, Guangxi Academy of Medical Sciences, The People's Hospital of Guangxi Zhuang Autonomous Region, Nanning, Guangxi Zhuang Autonomous Region, China

**Keywords:** benign stricture, case report, choledochectasia, cholelithiasis, cholestasis, double gallbladder, duodenal ulcer

## Abstract

Anatomical variants of the biliary system, such as a double gallbladder, are rare congenital anomalies. Although benign strictures of the duodenal bulb are common, their role as a long-term, subtle cause of biliary obstruction leading to complex pathological changes is rarely reported. This case report presents a complicated case in which a duodenal ulcer-related stricture caused chronic biliary obstruction, leading to cholestasis, dilation of the common bile duct (choledochectasia), and gallstone formation. These processes were further complicated by the presence of a double gallbladder anomaly, eventually requiring surgery. A 56-year-old male was admitted for abdominal pain. His medical history included right-sided nephrectomy, a duodenal ulcer, and previous embolization for duodenal hemorrhage. Imaging studies revealed gastric retention, significant dilation of intrahepatic and extrahepatic bile ducts accompanied by pneumobilia, and an incidental finding of a double gallbladder anomaly. One gallbladder exhibited evidence of cholestasis, while the other was impacted with stones. Gastroscopy confirmed a sever narrowing of the duodenal bulb. After failed conservative management, a multidisciplinary team performed a distal gastrectomy (Billroth II), double cholecystectomy, and choledochotomy with T-tube drainage. Surgical exploration confirmed a fibrotic duodenal stricture and sludge-filled gallbladders. The patient recovered uneventfully. This case illustrates that benign duodenal strictures can lead to chronic biliary obstruction, a process significantly aggravated by anatomical variants. This report emphasizes that unexplained biliary dilation accompanied by cholestatic laboratory findings should include proximal obstructive lesions, particularly duodenal bulb strictures, in the differential diagnosis. When such lesions coexist with rare anatomical variants like a double gallbladder, a complex pathological loop forms. Multidisciplinary, definitive surgery is often the solution when conservative therapy fails, offering new insights into the interaction between the upper and biliary digestive systems.

## 1 Introduction

Choledochectasia is a common clinical sign in hepatobiliary surgery, with a broad differential diagnosis typically focusing on choledocholithiasis, periampullary carcinoma, pancreatic cancer, biliary strictures, or congenital cysts (1). Duodenal bulb ulcers are also very prevalent, with common complications like hemorrhage, perforation, and obstruction (2). However, the complete pathological progression from post ulcer healing fibrotic stenosis, to a chronic proximal factor causing long-term biliary drainage impairment, eventually leading to progressive biliary dilation, cholestasis, and secondary lithogenesis, has rarely been systematically documented. Double gallbladder (DGB) is a rare congenital biliary anomaly with an incidence of approximately 1/4000. It originates embryologically from the duplication of the caudal branch of the hepatic diverticulum (3). While often asymptomatic, DGB can complicate cholelithiasis and cholecystitis and is associated with biliary anatomical variations, posing serious surgical challenges (4, 5). The coexistence of this rare anatomy with the aforementioned biliary pathology can create a unique, mutually aggravating clinical scenario.

This report details a complex case of chronic biliary obstruction, significant choledochectasia, and biliary lithiasis secondary to a duodenal bulb ulcer stricture, complicated by a double gallbladder anomaly. We aim to: (1) Raise awareness of benign duodenal strictures as a potential cause of biliary dilation; (2) Elucidate the “vicious cycle” of stasis, lithogenesis, and anatomical variation; and (3) Discuss the necessity of individualized surgical strategies within a multidisciplinary team (MDT) framework.

## 2 Case presentation

### 2.1 Patient information & chief complaints

A 56-year-old male was admitted on December 9, 2025, with a 10-day history of recurrent abdominal pain. The pain was severe, persistent, and poorly localized, without radiation. The patient had a history of “duodenal hemorrhage”: in February 2022, he underwent two embolization procedures involving the superior pancreaticoduodenal and gastroduodenal artery branches at our institution.

### 2.2 Medical history

The patient underwent a right nephrectomy in 2014 and reported no history of hypertension, diabetes, hepatitis, or tuberculosis. He denied long-term alcohol consumption or any known drug allergies. He also denied regular NSAID use and had no smoking history. He had no prior history of Helicobacter pylori infection. After the 2022 bleeding episode, he received intermittent proton pump inhibitor therapy with suboptimal compliance and no maintenance treatment. He reported no prior biliary symptoms (including right upper quadrant pain, jaundice, cholangitis, cholecystitis, or pancreatitis). Despite gastric retention, he experienced only mild weight loss (~2 kg over 3 months) and maintained a body mass index of 19.0 kg/m^2^ (height 170 cm, weight 55 kg), without overt malnutrition. No long-term medications other than intermittent PPI use were taken, and he was not on anticoagulants or antiplatelet agents.

### 2.3 Clinical findings

His vital signs were stable on admission. Physical examination revealed no evidence of jaundice or pallor. The abdomen was soft, with no rebound tenderness, and Murphy's sign was **negative**.

### 2.4 Laboratory tests

Liver function tests showed a normal bilirubin profile with total bilirubin measured at 8.8 μmol/L and, direct bilirubin measured at 1.7 μmol/L. However, serum bile acids were elevated at 65 μmol/L, indicating cholestasis without overt obstructive jaundice. Tumor markers (CA19-9, CEA, etc.) were within normal limits.

## 3 Imaging findings

Imaging findings are shown in Figure 1.

**Figure 1:**
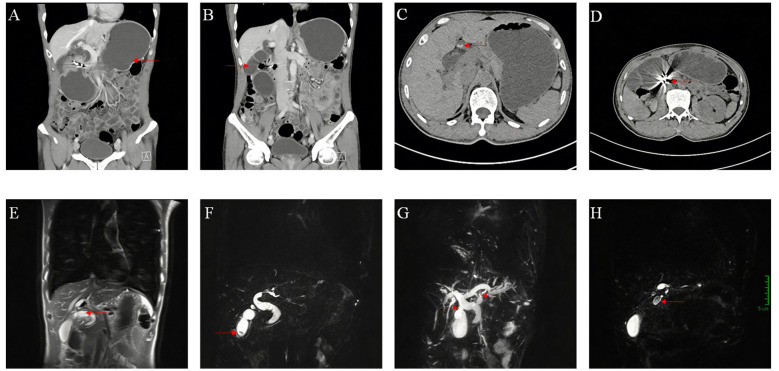
Computed tomography (CT) and magnetic resonance cholangiopancreatography (MRCP) findings. CT images **(A–D)**: **(A)** Gastric retention (dilated stomach with food residue, red arrow); **(B)** The main gallbladder (collapsed, red arrow); **(C)** Gallstones within the accessory gallbladder; **(D)** Embolization coils (curvilinear high-density artifacts) from prior duodenal hemorrhage treatment. MRCP images **(E–H)**: **(E)** Common bile duct (CBD) dilation with measured diameter of approximately 15 mm (red arrow); **(F)** The main gallbladder contains sand-like stones; **(G)** Two separate cystic ducts (red arrows) originating from the main and accessory gallbladders; **(H)** Accessory gallbladder containing multiple stones (red arrow).

### 3.1 Ultrasound

Initial ultrasonography showed dilation of the common Bile duct (CBD) and the left intrahepatic duct.

### 3.2 MRCP

Magnetic resonance cholangiopancreatography confirmed diffuse intrahepatic and extrahepatic biliary dilation (CBD diameter 15 mm). An additional gallbladder was identified in segment IV (left lobe), filled with stones. Possible obstruction at the distal CBD or ampulla was suspected.

### 3.3 Contrast-enhanced CT

Computed tomography with contrast showed significant biliary dilation with pneumobilia (diameter 1.5 cm) and intrahepatic stones. The gallbladder was distended with intraluminal gas. Notably, curvilinear high-density structures were noted in the duodenal descending segment, consistent with prior embolization coils.

### 3.4 Endoscopy

Gastroscopy revealed massive gastric retention and a severely stenotic duodenal bulb impassable to the endoscope. A barium contrast study further confirmed luminal narrowing and deformation.

## 4 Diagnosis and treatment process

Primary Diagnosis:

- Cicatricial stenosis secondary to Duodenal bulbar ulcer

Secondary/Associated Diagnoses:

- Secondary intrahepatic and extrahepatic bile duct dilation with pneumobilia
- Chronic calculous cholecystitis (gallstone-filled gallbladder)
- Double gallbladder anomaly (supernumerary gallbladder)
- Gastric retention

Given the patient‘s presentation with biliary dilation (CBD 15 mm) without overt jaundice (normal bilirubin but elevated serum bile acids), pneumobilia, gastric retention, and a double gallbladder anomaly, a broad differential diagnosis was systematically considered (Table 1).

**Table 1:** Differential diagnosis of biliary dilation in this patient.

Diagnosis	Evaluation and exclusion
Choledocholithiasis	MRCP showed no large obstructing stone; intraoperatively.
Malignancy	Contrast-enhanced CT and MRCP revealed no mass at the ampulla. Tumor markers (CA19-9, CEA) were normal. Intraoperative palpation and inspection of the periampullary region (as much as permitted by fibrosis) showed no neoplasm.
Benign distal CBD stricture	No focal stricture of the CBD was seen on MRCP. The biliary dilation was smooth and tapering, not abrupt.
Ectopic biliary drainage	No ectopic opening of the CBD into the duodenal bulb.
Sphincter of Oddi dysfunction	This could not be directly measured, but the presence of chronic duodenal inflammation and dense periduodenal scarring made secondary sphincter dysfunction plausible. However, no prior biliary intervention or manometry was available to confirm a primary sphincter disorder.
Post-interventional pneumobilia	The patient had no prior ERCP, sphincterotomy, or biliary surgery. The only prior intervention was arterial embolization for duodenal bleeding (no biliary manipulation). Thus, pneumobilia was attributed to duodeno-biliary reflux from the duodenal stricture rather than iatrogenic causes.
Primary double gallbladder-associated bile stasis	While the double gallbladder anomaly may predispose to stasis (due to uncoordinated emptying), it alone cannot explain the marked CBD dilation (15 mm) or pneumobilia. The intraoperative finding of dense adhesions between the scarred duodenal bulb and the porta hepatis strongly implicated the stricture as the primary mechanical factor.

### 4.1 Therapeutic interventions

Initial conservative management including fasting, Proton Pump Inhibitor, decompression failed to resolve the mechanical obstruction. After a multidisciplinary discussion involving the hepatobiliary, pancreatic, and spleen surgery department, the imaging department, the gastroenterology department, and the interventional department, it was determined that the duodenal stricture as the primary etiology, causing both gastric outflow obstruction and secondary biliary hypertension. Based on the gastroscopy results, since it is impossible to pass through the narrow area, echo-endoscopy (EUS) and endoscopic retrograde cholangiopancreatography (ERCP) are not considered. Percutaneous transhepatic biliary drainage (PTBD) was considered as a temporizing measure but was not pursued for the following reasons: It would only address biliary obstruction without resolving the duodenal stricture or gastric retention; The patient had no acute cholangitis and normal bilirubin, so urgent biliary decompression was not required; Given the need for definitive treatment of both gastric outlet and biliary obstruction, a definitive operation was preferred over a staged approach with PTBD followed by surgery.

On December 24, 2025, the patient underwent an exploratory laparotomy. Intraoperative findings revealed dense adhesions between the fibrotic, thickened duodenal bulb and the porta hepatis, confirming the preoperative diagnosis of cicatricial stenosis secondary to chronic ulceration. Additionally, the surgery also confirmed the presence of a double gallbladder anomaly, consisting of a distended main gallbladder measuring approximately 8 × 3 cm and a left-sided supernumerary gallbladder measuring 4 × 2 cm, both of which were filled with stones (Figure 2). The liver exhibited mild cholestatic changes, consistent with chronic biliary obstruction. Due to the extensive fibrosis and severe anatomical distortion of the duodenum and perihilar region, simple biliary drainage alone was deemed insufficient. Therefore, a distal subtotal gastrectomy and duodenal bulbectomy with Billroth II reconstruction was performed to definitively bypass the duodenal stricture and restore gastrointestinal continuity. No biliary bypass was created because the intraoperative finding was no fixed, intrinsic stricture of the CBD. This was combined with double cholecystectomy and CBD exploration with T-tube insertion to clear the biliary tree of sand-like stones and establish external biliary drainage. The operation proceeded smoothly without intraoperative complications.

**Figure 2:**
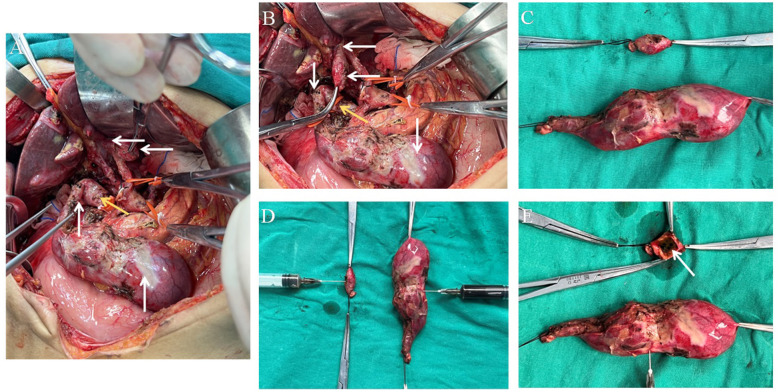
Intraoperative images. **(A)** & **(B)** As shown by the white arrow, the vertical arrow points to the main gallbladder and its cystic duct, while the horizontal arrow points to the accessory gallbladder and its cystic duct. The accessory cystic duct does not merge into the common bile duct but instead originates from the left liver. The yellow arrow represents the dilated common bile duct; **(C)** Both gallbladders possess a complete gallbladder anatomical structure; **(D)** Both gallbladders had bile-like fluids extracted; **(E)** An examination of the accessory gallbladder revealed several small black stones.

### 4.2 Histopathologic examination of the resected specimens

Histopathologic examination of the resected stomach revealed chronic non-atrophic gastritis with mild foveolar hyperplasia and no significant inflammatory activity. No intestinal metaplasia, dysplasia, or malignancy was identified. The gastric wall architecture was preserved, and there was no evidence of Helicobacter pylori organisms on special staining. Duodenal bulb stricture: Microscopic examination showed dense fibrous tissue replacing the muscularis mucosae and submucosa, with chronic inflammatory cell infiltration and no evidence of dysplasia or malignancy. The features were consistent with benign cicatricial stenosis secondary to chronic ulceration. Both gallbladders showed chronic cholecystitis with mural fibrosis, Rokitansky-Aschoff sinuses, and mixed pigment/cholesterol stones. No epithelial dysplasia or carcinoma was identified. The accessory gallbladder also exhibited similar chronic inflammatory changes.

These findings confirm the benign nature of the stricture and exclude occult malignancy.

### 4.3 Outcomes & follow-up

The patient achieved an uneventful postoperative recovery with no significant complications, and the T-tube remained patent and well-functioning. Abdominal pain was alleviated compared with the preoperative status. The patient was discharged with the T-tube in place on postoperative day 10.

At the 6-week follow-up, the patient reported complete resolution of abdominal pain and had resumed a normal diet. Following confirmation of biliary tract patency by T-tube cholangiography, the T-tube was successfully removed. The long-term follow-up after the surgery, lasting for 6 months, confirmed that the patient's liver function was normal, there was no recurrence of biliary tract or gastric outlet symptoms, no nutritional complications, and no adverse events in the later stage. The clinical timeline is shown in Figure 3.

**Figure 3:**
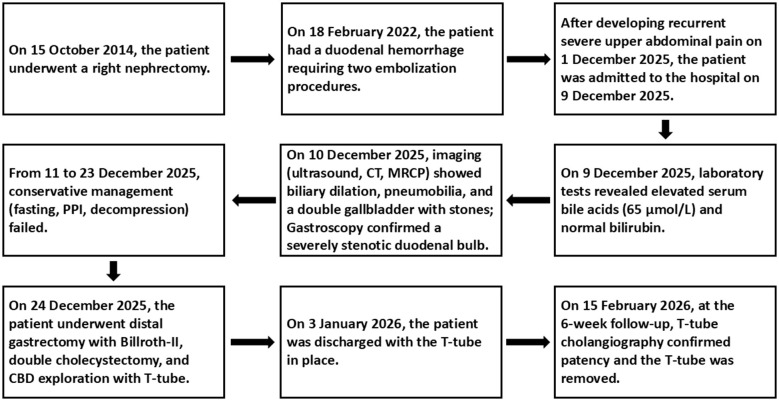
The clinical timelines.

## 5 Discussion

This case presents a unique disease sequence initiated by a duodenal ulcer-related stricture and complicated by a congenital biliary anomaly. It highlights a frequently overlooked deep connection between benign upper gastrointestinal lesions and biliary pathology.

The mechanism by which a benign duodenal lesion induces biliary obstruction is likely multifactorial. Based on the intraoperative and imaging findings, we propose a multifactorial mechanism as a likely explanation for how the duodenal bulbar stricture contributed to biliary obstruction. First, persistent inflammation and scarring of the duodenal bulb lead to significant wall thickening and narrowing of the lumen. Because the distal CBD lies in close anatomical proximity to the medial wall of the duodenal bulb, this fibrotic process can directly compress the bile duct or create a kinking effect, ultimately resulting in mechanical obstruction (6, 7). Second, chronic duodenitis may extend to the ampullary region, causing inflammatory edema or even fibrosis of the Sphincter of Oddi, which might consequently contribute to a functional component of obstruction (8, 9). Third, the presence of pneumobilia on preoperative CT imaging provides direct evidence of duodeno-biliary reflux. Under conditions of elevated intraduodenal pressure secondary to the stricture, enteric contents such as gas and bacteria are forced retrogradely into the biliary tract (10, 11). This phenomenon not only explains the risk of cholangitis but may also create an intraluminal “gas-lock” effect, further exacerbating the outflow obstruction. It is important to note that the bile duct itself did not harbor a primary fibrotic stricture; the dilation was secondary to extrinsic duodenal compression and functional outflow impairment. Therefore, duodenal bulbectomy alone was sufficient to relieve the biliary obstruction. The T-tube cholangiogram at 6 weeks confirmed a widely patent CBD without residual obstruction.

This chronic, incomplete biliary obstruction inevitably leads to bile stasis and concentration resulting in cholestasis. This condition creates a favorable physicochemical environment for the precipitation of bilirubin or cholesterol, ultimately leading to lithogenesis (12). The “muddy” or sludge-like stones, intraoperatively encountered, within the CBD and left hepatic duct are the direct products of this prolonged biliary stasis. Importantly, this pathophysiological process occurred in the presence of a double gallbladder anomaly, which adds a critical dimension to the case. The presence of a supernumerary gallbladder may inherently disrupt the rhythmic storage and ejection of bile, predisposing the system to stasis even under physiological conditions. When biliary pressure increase under pathological condition, both gallbladders act as passive “reservoirs” for stagnant bile, potentially accelerating the process of stone formation. Moreover, this rare anatomical variant—in which the accessory cystic duct was observed to enter the left hepatic duct—has been documented in only a few previous reports (13)—significantly increases the difficulty of preoperative diagnosis and surgical planning. From an embryological perspective, this configuration aligns with the classification system proposed by Harlaftis, which distinguishes between *vesica fellea divisum* (split primordium) and *vesica fellea duplex* (accessory gallbladder), with the present case representing a subtype of the latter (14).

From a clinical standpoint, this case offers several important insights. First, it underscores the need for diagnostic vigilance when confronted with “idiopathic” biliary dilation in the absence of jaundice but with biochemical evidence of cholestasis, such as elevated bile acids. In these situations, the differential diagnosis must be broadened to include proximal lesions of the duodenal bulb or descending duodenum. Although, magnetic resonance cholangiopancreatography (MRCP) is valuable for assessing biliary anatomy, upper gastrointestinal endoscopy remains the gold standard for directly visualizing mucosal strictures that may escape cross-sectional imaging. Second, the failure of conservative management in this case highlights the fundamental reality that organic, fibrotic strictures are unlikely to respond to pharmacological intervention alone. While endoscopic approaches such as endoscopic retrograde cholangiopancreatography (ERCP) with stent placement may offer a temporary relief biliary obstruction, they cannot spontaneously address the concomitant gastric outlet obstruction or the presence of anomalous gallbladders. The definitive surgical approach performed in this patient—distal subtotal gastrectomy and duodenal bulbectomy with Billroth II reconstruction combined with double cholecystectomy and CBD exploration with T-tube drainage—was curative in nature. This approach simultaneously bypassed the duodenal stricture, resolved the gastric retention, cleared the biliary tree of stones, and removed the future risk posed by the anomalous, stone-laden gallbladders. This comprehensive strategy exemplifies the surgical principle of addressing both the root cause and its downstream consequences in complex, multiorgan pathologies.

A review of the existing literature underscores the uniqueness of the present case. While there are isolated reports of duodenal ulcer perforation resulting in bilioenteric fistulae, and others documenting the association between periampullary diverticula and biliary disease, a case in which a non-perforated, scarring duodenal ulcer gradually over several years—induces progressive biliary dilation, cholestasis, secondary lithogenesis, and coexists with a double gallbladder anomaly has not, to our knowledge, been systematically described in the English-language literature.

Consequently, this case provides valuable documentation of this specific and instructive pathophysiological chain, offering a new perspective into the complex relationship between common upper gastrointestinal disorders and the biliary system. However, longer prospective follow-up would be valuable to confirm the sustained benefit of this surgical approach.

## 6 Conclusion

This report details a complex clinical case of secondary biliary dilation and lithiasis caused by a duodenal bulb ulcer stricture, further complicated by a double gallbladder anomaly. It demonstrates that benign duodenal strictures can act as a persistent force causing severe biliary pathology through both mechanical compression and inflammatory processes. When such situations coexist with rare anatomical variations, the complexity of diagnostic and therapeutic challenges increases substantially. The successful management of this case highlights the necessity of thorough upper gastrointestinal evaluation and the potential need for radical, multidisciplinary surgery to achieve optimal outcomes.

## 7 Patient perspective

I am a 56-year-old man who has always considered myself relatively healthy, aside from a right kidney removal years ago. In early December 2025, I began experiencing recurrent, severe pain in my upper abdomen. The pain was constant and made it difficult to eat or even sleep. I went to the hospital, where doctors ordered blood tests and scans. They found that my bile ducts were abnormally widened and that I had an unusual anatomical variation—two gallbladders instead of one.

What worried me most was that I also had a long-standing duodenal ulcer that had left a tight scar, narrowing the outlet of my stomach. Despite medications and fasting, my symptoms did not improve. The medical team explained that the scarred duodenum was blocking not only food passage but also the normal flow of bile, leading to backup and stone formation in both gallbladders.

I agreed to a major surgery that removed the narrowed part of my duodenum, both gallbladders, and explored my common bile duct. The operation took several hours, but I woke up with no pain. I stayed in the hospital for 10 days with a temporary drainage tube. 6 weeks later, the tube was removed after confirming that my bile ducts were clear. Today, I can eat normally without any abdominal pain. I am grateful to the multidisciplinary team for solving a problem that had been silently damaging my digestive system for years.

## Data Availability

The original contributions presented in the study are included in the article/supplementary material, further inquiries can be directed to the corresponding author.
